# The importance of wildlife in the ecology and epidemiology of the TBE virus in Sweden: incidence of human TBE correlates with abundance of deer and hares

**DOI:** 10.1186/s13071-018-3057-4

**Published:** 2018-08-29

**Authors:** Thomas G. T. Jaenson, Erik H. Petersson, David G. E. Jaenson, Jonas Kindberg, John H.-O. Pettersson, Marika Hjertqvist, Jolyon M. Medlock, Hans Bengtsson

**Affiliations:** 1 Department of Organismal Biology, Uppsala University, Norbyvägen 18d, SE-752 36 Uppsala, Sweden; 2 Department of Aquatic Resources, Division of Freshwater Research, Swedish University of Agricultural Sciences, Stångholmsvägen 2, SE-178 93 Drottningholm, Sweden; 3 Department of Automatic Control, Lund University, SE-221 00 Lund, Sweden; 4 Department of Wildlife, Fish and Environmental Studies, Swedish University of Agricultural Sciences, SE-901 83 Umeå, Sweden; 5 Department of Infectious Disease Epidemiology and Modelling, Norwegian Institute of Public Health, Lovisenberggata 8, N-0456 Oslo, Norway; 6 Department of Medical Biochemistry and Microbiology (IMBIM), Zoonosis Science Center, Uppsala University, Uppsala, Sweden; 7 Marie Bashir Institute for Infectious Diseases and Biosecurity, Charles Perkins Centre, School of Life and Environmental Sciences and Sydney Medical School, the University of Sydney, Sydney, New South Wales 2006 Australia; 8 Public Health Agency of Sweden, Nobels väg 18, SE-171 82 Solna, Sweden; 9 Medical Entomology Group, Emergency Response Department, Public Health England, Porton Down, Salisbury, UK; 10 Health Protection Research Unit in Emerging Infections & Zoonoses, Porton Down, Salisbury, UK; 11 Swedish Meteorological and Hydrological Institute (SMHI), Gothenburg, Sweden

**Keywords:** *Ixodes ricinus*, *Capreolus capreolus*, *Cervus elaphus*, *Lepus*, Hare, Red deer, Roe deer, Tick-borne encephalitis, TBE virus, Climate change

## Abstract

**Background:**

Tick-borne encephalitis (TBE) is one tick-transmitted disease where the human incidence has increased in some European regions during the last two decades. We aim to find the most important factors causing the increasing incidence of human TBE in Sweden. Based on a review of published data we presume that certain temperature-related variables and the population densities of transmission hosts, i.e. small mammals, and of primary tick maintenance hosts, i.e. cervids and lagomorphs, of the TBE virus vector *Ixodes ricinus*, are among the potentially most important factors affecting the TBE incidence. Therefore, we compare hunting data of the major tick maintenance hosts and two of their important predators, and four climatic variables with the annual numbers of human cases of neuroinvasive TBE. Data for six Swedish regions where human TBE incidence is high or has recently increased are examined by a time-series analysis. Results from the six regions are combined using a meta-analytical method.

**Results:**

With a one-year time lag, the roe deer (*Capreolus capreolus*), red deer (*Cervus elaphus*), mountain hare (*Lepus timidus*) and European hare (*Lepus europaeus*) showed positive covariance; the Eurasian elk (moose, *Alces alces*) and fallow deer (*Dama dama*) negative covariance; whereas the wild boar (*Sus scrofa*), lynx (*Lynx lynx*), red fox (*Vulpes vulpes*) and the four climate parameters showed no significant covariance with TBE incidence. All game species combined showed positive covariance.

**Conclusions:**

The epidemiology of TBE varies with time and geography and depends on numerous factors, i.a. climate, virus genotypes, and densities of vectors, tick maintenance hosts and transmission hosts. This study suggests that the increased availability of deer to *I. ricinus* over large areas of potential tick habitats in southern Sweden increased the density and range of *I. ricinus* and created new TBEV foci, which resulted in increased incidence of human TBE. New foci may be established by TBE virus-infected birds, or by birds or migrating mammals infested with TBEV-infected ticks. Generally, persistence of TBE virus foci appears to require presence of transmission-competent small mammals, especially mice (*Apodemus* spp.) or bank voles (*Myodes glareolus*)*.*

**Electronic supplementary material:**

The online version of this article (10.1186/s13071-018-3057-4) contains supplementary material, which is available to authorized users.

## Background

### The great medical importance of *Ixodes ricinus*

In Europe *Ixodes ricinus* is the most important vector of viruses, bacteria and protozoa, which potentially can cause disease in humans, companion animals or livestock. This tick species is a proven or putative vector of > 25 different viruses and microorganisms, which are known or highly suspected to be potential pathogens of humans [1–9]. In several European countries the numbers of diagnosed cases of tick-borne diseases transmitted by *I. ricinus* to humans have increased during the last two decades [10–19], but in some regions disease incidence appears not to have increased [20] or has even declined [21]. Tick-borne encephalitis (TBE) and Lyme borrelioses (LB, Lyme disease) are two disease complexes of major medical importance in the Palaearctic and Holarctic regions, respectively. The etiological agents of both complexes are vectored mainly by *Ixodes* ticks. In most parts of Europe *I. ricinus* is the main vector of the TBE virus and the *Borrelia burgdorferi* (*s.l*.) spirochaetes causing LB. In the following text, “tick” and “ticks” denote *I. ricinus*.

In this paper we will analyse why the incidence of human TBE in Sweden has increased during the last two decades focussing on the potential impact of wildlife and climate.

### Risk of infection

A high relative humidity (≥ *c.*85%) at ground level in the microhabitat is a prerequisite for the tick population’s survival [22]. The amount of precipitation will therefore influence tick density and geographical distribution. Tick development rate and questing activity are strongly affected by temperature. The life-cycle can be completed in two years in habitats with exceptionally warm temperatures during the tick season [22]. The density of TBEV-infected nymphs in the habitat is a major determinant of the risk that people will be infected by TBEV. The other variable is the degree of human exposure to TBEV-infected nymphs. As such, if there is a certain density of infected nymphs in a particular habitat, increased activity in that habitat of unvaccinated people or of people ignorant about tick-transmitted infections will increase their risk. However, people who are dressed properly (wearing boots, trousers tucked inside the boots, usage of tick repellents, etc.) when visiting tick-infested areas will be less exposed to potential vector ticks and will thus reduce their risk of infection. The TBEV transmission intensity in time and space depends on the infection prevalence in the tick population, the density of host-seeking TBEV-infective ticks and on the availability, i.e. the density and activity of susceptible tick hosts and reservoir-competent tick hosts. The density of potential vector ticks, i.e. mainly nymphs, is dependent on the population densities of different tick maintenance hosts in preceding years. Such tick host species are the roe deer (*Capreolus capreolus*), other cervids and hares. But the actual vector density is also, to a great extent, dependent on the previous season’s availability of small mammals and, to a lesser extent, on ground-feeding birds to host-seeking tick larvae. The TBEV infection prevalence in the tick population will be related to the availability to host-seeking immature ticks of transmission-competent vertebrates, i.e. mainly several species of small mammals [3], particularly *Apodemus* mice [3, 23] and on several species of ground-frequenting birds [3]. This is not only because these smaller vertebrates may be transmission competent but also because they are generally the main hosts for tick larvae [24, 25].

### Ungulates: promoters of tick reproduction and human disease

Roe deer is now a common sight in many gardens and urban parks in the cities and towns of southern and central Sweden. Even wild boars (*Sus scrofa*) have begun to visit the city centres of southern Sweden. There is significantly more wildlife in most European countries, including Sweden, today compared to fifty years ago [26, 27]. This change in size and composition of the fauna of browsing and grazing large mammals has been accompanied by extensive changes in the plant communities. Additionally, in some Swedish municipalities intentional breeding of deer has been promoted. The increased biomass of medium and large wildlife species in Sweden during recent decades [26] has increased the availability of blood for all active tick stages and has also provided an increased density of optimal mating sites for the adult ticks.

Permanent populations of *I. ricinus* depend on the long-term presence of relatively large vertebrate hosts such as deer, cattle, hares and dogs. This is because the adult *I. ricinus* females rarely feed on small mammals or on ground-frequenting birds. In a similar manner, adult males of *I. ricinus* appear to avoid infesting small vertebrates [25] but prefer medium or large vertebrates as mating sites. Here, the sexually appetitive tick males are likely to encounter females of their own species.

Some species of cervids, such as roe deer, are frequently found in deciduous broad-leaved woodlands or mixed deciduous broad-leaved spruce woodlands as well as in the ecotones between such biotopes and adjacent meadows or agricultural fields. Such biotopes are optimal also for *I. ricinus* [28]. Thus, roe deer and other cervid species are in many ways beneficial to *I. ricinus*.

The large wild herbivores are important ecosystem components, which provide many ecosystem services to humans. However, the negative effects that cervids indirectly may have on human health are considerable [29, 30] yet occasionally dismissed [31, 32]. It is therefore important to reiterate certain facts about some of the undesirable impacts on human health that roe deer and some other cervid species may have when they are present within or close to areas inhabited by humans.

Several investigations in Europe and North America have shown that cervids, even at very low densities [33–35], are important drivers of tick abundance. Cervids are an important source of blood to all active stages of *I. ricinus*, in particular to the adult tick females [25, 36]. Massive infestations of > 2000 ticks on one individual roe deer are on record [25, 36]. The smaller roe deer seems, compared to the larger red deer, to be more attractive or more readily available for *I. ricinus* [37]. Cervids are important tick assembly and mating sites where sexually appetitive tick males can search for and inseminate tick females. Indirectly, deer are important tick producers since they feed large numbers of adult ticks [25]. Cervids also serve as moving objects for the passive, short- and long-distance spread of ticks potentially infected with viruses, bacteria and protozoa that may be pathogenic to humans or domesticated mammals [29, 38–42]. Moreover, roe deer are themselves suspected or proven reservoirs of pathogens and parasites of medical or veterinary importance [29, 43–49] such as *Rickettsia helvetica* [48, 50, 51], *Bartonella schoenbuchensis* [50, 52], *Anaplasma phagocytophilum* [48, 50], *Babesia venatorum* [50, 53, 54] and bot fly larvae (*Cephenemyia* spp.) [51, 55, 56].

In Sweden, roe deer numbers peaked in the early 1990s; main reasons were (i) reduced predation of roe deer due to high mortality of the red fox (*Vulpes vulpes*) population, caused by a scabies epizootic; (ii) low density of lynx (*Lynx lynx*); (iii) a series of mild winters, which increased overwintering survival in young deer; (iv) clear-felling of forests and abandonment of farmland, both of which then provided easily accessible nutrient-rich abandoned field vegetation, i.e. suitable food for wild ungulates [27, 38]; and (v) due to increased hunting of Eurasian elk (moose; *Alces alces*) since 1982 this population was drastically reduced, which consequently reduced competition for food between elk and roe deer [57].

Among wildlife on the Scandinavian Peninsula the roe deer is not the only potentially important maintenance host of *I. ricinus*. In Sweden, the populations and geographical ranges of red deer, fallow deer (*Dama dama*) and wild boar have also increased to unprecedented densities in recent years [58, 59]. In Scotland [60] and Norway [61, 62], the red deer is an important tick maintenance host. As such, cervids are producers or promoters of tick numbers. Tick infestation rates on wild boar, sheep (*Ovis aries*) and cattle (*Bos taurus*) seem to be much lower [33, 37] but in certain humid biotopes, with a thick litter layer, both cattle and sheep can sustain dense tick populations [33, 37]. Thus, presence of deer seems to be the main cause of the increased incidences of tick-borne diseases (TBDs) in humans in many European countries and the USA during recent decades [38–40, 60, 63–78].

### Small mammals, hosts of tick larvae and transmission hosts of TBEV

In Sweden shrews (Soricidae) and rodents (Muroidea) are the main hosts for larval *I. ricinus* [25, 79, 80] while most tick nymphs feed on hares, cervids [25, 79] and birds [81]. The TBEV infection prevalence in the nymphs and adult ticks is mainly related to the availability to host-seeking immature ticks of transmission-competent vertebrates, mainly small mammals, particularly *Apodemus* spp. [9, 82, 83] and voles [3]. A main determinant of the numbers of rodents is the availability of nutrients, which is partly related to the production (masting) of fruits of oak, beech, chestnut and other trees [24]. These annual variations in the density of TBEV-infected nymphs will potentially affect the human TBE incidence. Abundance estimates for small mammals in our study regions in southern Sweden were not available and could therefore, unfortunately not be included in our analyses. In Sweden, important hosts for tick larvae, and to a lesser degree for nymphs, are the yellow-necked field mouse (*Apodemus flavicollis*) and the wood mouse (*A. sylvaticus*) [25]. These rodent species are most likely important for the transmission of TBEV among immature ticks [9, 24, 83–91]. The varying densities of the small mammals likely constitute a substantial part of the unexplained contribution to the TBE incidence in humans. Support for this comes from the research of Bespyatova and co-workers [92] who studied the population dynamics of small mammals in a TBEV-enzootic region in Karelia during 1995–2003. They found that the bank vole is the main host for > 60% of the larvae and nymphs of *I. persulcatus* and for > 60% of all active tick stages of *I. trianguliceps*, and that the activity of a TBEV focus is mainly determined by the density of older individuals of bank voles. Similarly, in the USA Ostfeld and co-workers [93, 94] found that the risk for people to become exposed to *I. scapularis* nymphs infected with *B. burgdorferi* is significantly correlated with the density of the white-footed mouse (*Peromyscus leucopus*). This rodent is a key reservoir and transmission host of the Deer tick virus (which is one of the two lineages of the Powassan virus - a member of the tick-borne encephalitis virus complex), *B. burgdorferi*, *B. miyamotoi*, *A. phagocytophilum* and *Babesia microti* [94, 95]. In Europe *Apodemus* mice seem to have a comparable key role in the disease ecology of TBEV [85], *B. afzelii* [96], *B. bavariensis* [97], *B. miyamotoi* and “*Candidatus* Neoehrlichia mikurensis” [98].

In analogy with the view of van Wieren & Hofmeester [33] regarding the density of *Borrelia-*infected nymphs, the density of TBEV-infected nymphs in a TBEV focus will generally be the outcome of the (i) density of tick maintenance hosts (usually deer), which indirectly play the greatest role in determining the reproductive success of the adult ticks, i.e. how many tick larvae that will be produced; and (ii) availability of rodents, especially *Apodemus* spp. and the bank vole (*Myodes glareolus*), and shrews, which have a great impact on the feeding success and survival of the tick larvae, and also on the TBEV transmission rate to the tick larvae. Certainly, the densities of other potential tick hosts and the climate and weather will also affect the density of infected nymphs. However, since small mammals are generally the main hosts for tick larvae [25, 34], at least in southern Sweden, it is presumably the availability of small mammals, rather than that of deer, which has the greatest impact on density of infected nymphs and on the variation in TBE incidence among different years.

### TBE in Sweden

**Fig. 1:**
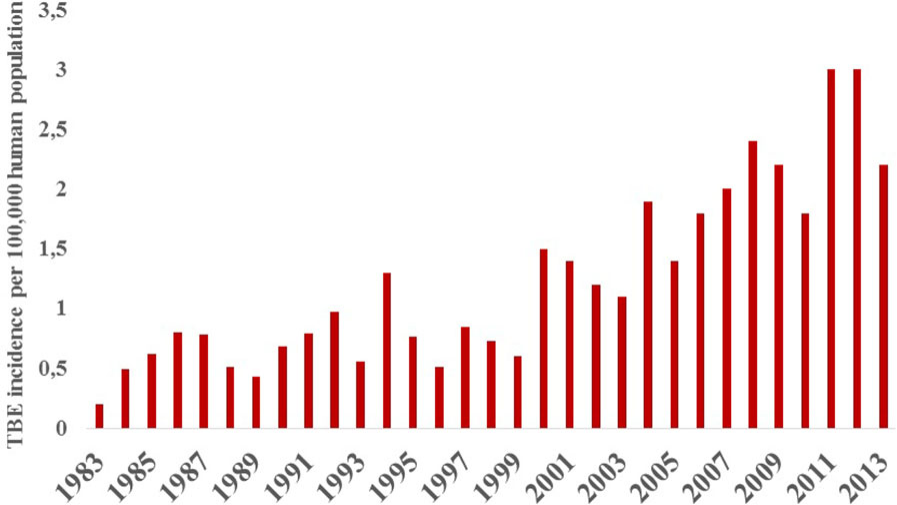
Annual incidence of neuroinvasive TBE per 100,000 inhabitants in Sweden for the 31-year period 1983–2013. Data recorded by the Public Health Agency of Sweden, formerly Swedish Institute for Communicable Disease Control (SMI), Solna, Sweden

The first human TBE case in Sweden was described in 1954 [99–101]. Since the 1980s the annual incidence of human TBE has increased almost continuously. In the 1990s, there were about 60–80 cases/year, except in 1994 when 114 cases were recorded [102]. Since the year 2000, there have been > 100 cases reported annually (Fig. 1). In 2011 and 2012, > 280 cases were diagnosed each year [40, 102]. Trend analysis of the incidence of human TBE cases reveals a significantly increasing trend of 6% per year during 1983–2016 [103].

A main reason for the increasing incidence of TBE is considered to be that the density and distribution of the main TBEV vector, *I. ricinus*, have increased. From the early 1980s to 2008 *I. ricinus* increased its estimated range by 10% in Sweden and in the area of Sweden north of 60 °N the tick’s range more than doubled from 12.5% to 27% of the area [38]. Data also suggest that the number of *I. ricinus* ticks in southern and central Sweden increased markedly during the last ~30 years [38].

**Fig. 2:**
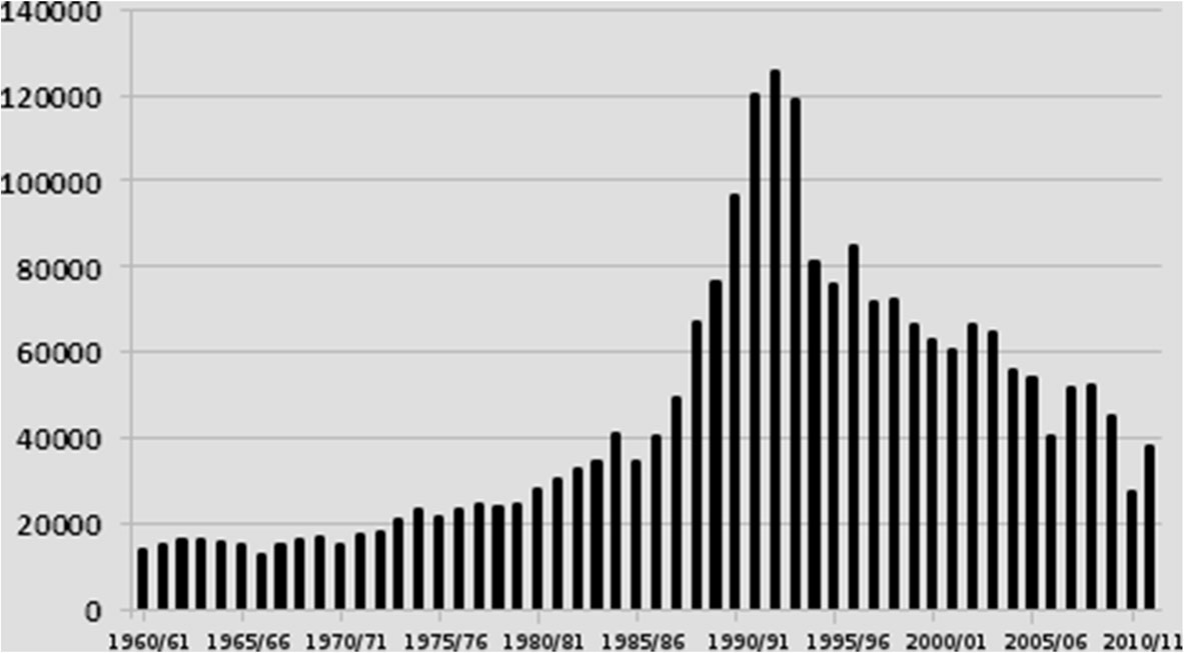
Total numbers of roe deer shot (y-axis) each hunting year* during 1960/61–2011/12 (x-axis) in six Swedish counties (Stockholms, Uppsala, Södermanlands, Östergötlands and Västra Götalands län), which have the highest incidences of human TBE in Sweden, and Skåne län, with a low TBE incidence but where several new TBE foci have appeared during the last decade. *Most hunting activities take place during the autumn

The most likely reasons for its increased abundance and range in Sweden were reviewed by Jaenson et al. [38, 40] and Medlock et al. [39, 104]. They concluded that several factors, including increased abundance of tick maintenance hosts, and changes of the climate and vegetation have interacted to facilitate the increased tick abundance in Sweden [38–40, 104]. The two most important drivers appear to have been the high availability of cervids, particularly roe deer (Fig. 2; [38, 60, 74]) and the milder climate [105, 106]. The increased densities of other medium and large mammals have presumably also contributed to the increased transmission of TBEV.

### Climate change and the incidence of tick-borne diseases

In northern Europe, climate change has been linked to an increasingly longer vegetation period and, in most regions of Scandinavia, increased annual precipitation. *Ixodes ricinus* and the pathogens of humans transmitted by this tick species are certainly highly sensitive to changes in temperature [106–108]. But climate change is only one among several factors, which may have affected the increasing incidence of several tick-borne infections during the last decades [76, 108, 109]. This is particularly so in the core area of the geographical range of *I. ricinus* where the changing climate has been less important for the increased incidence of human TBE and human LB than have changes in land use, animal host communities, human living conditions and societal factors. Thus, the geographical distribution and true density of *I. ricinus* are determined not only by availability of hosts, weather and climate but also by several other factors, some of which are interrelated [38, 39, 74, 105, 107].

In strong contrast to the relatively weak effect of climate change in the centre of the organism’s range is the marked effect of climate change in the peripheral areas. Thus, at the northern edges of the geographical range of *I. ricinus* climate change has had and will have quite distinct effects. Here, the warmer climate has permitted and will continue to permit *I. ricinus* [38, 61, 105] and *I. persulcatus* [110, 111] to extend their North-European population ranges further to the north. This is likely to increase the incidence of human infections associated with these tick species. A similar situation is true for *I. ricinus* at high altitudes in central Europe [112] and for *I. scapularis* and LB in Canada [113].

A warmer winter climate may additionally be beneficial to the tick hosts and for the ticks themselves, and could therefore increase the potential for TBEV transmission. Moreover, a prolonged vegetation period, i.e. more days with ambient temperatures > 5 °C, would likely increase winter survival of deer, hares, rodents and insectivores, and would also prolong the seasonal activity period of the ticks and therefore increase the opportunities for adult ticks, nymphs and larvae to host-seek and blood-feed. The result would be increased density and activity (= availability) of infective ticks, which could increase the risk that they would transmit TBEV to potential transmission hosts (small mammals, hares and birds) and to humans [101, 105].

Nymphs of *I. ricinus* in Sweden usually do not quest when the air temperature at ground level is below 5 °C. At temperatures > 9 °C some larvae of *I. ricinus* become active. Thus, at temperatures > 9 °C both nymphs and larvae may quest simultaneously and attach to the same individual small mammals, especially *Apodemus* spp., on which non-viraemic TBEV transmission from infective nymphs to susceptible larvae may occur. Therefore, one hypothesis would be that more days with temperatures > 9 °C should lead to increased frequency of co-feeding, which would lead to increased virus transmission. Such warmer periods should therefore increase the number of infective ticks and would therefore increase the risk for people to contract TBE.

### Research aims

The aims of this work are to analyse the ecology and epidemiology of TBEV in Sweden and then, based on this analysis, to evaluate the relative importance of the potentially most important factors, presumed to affect the incidence of human TBE in Sweden. We analysed statistically the potential effects of four temperature-related variables [the annual length in days of the vegetation period; the annual numbers of days with temperatures > 9 °C; the annual mean temperature (°C); and the annual (1969–2013) mean temperature deviation from the ‘normal mean temperature’], and of the annual abundances of the most important *I. ricinus* maintenance host species and their two most important predators, on the annual incidence of human neuroinvasive TBE in six TBE-endemic areas of southern Sweden.

## Methods

### Annual incidence of human neuroinvasive TBE

**Fig. 3:**
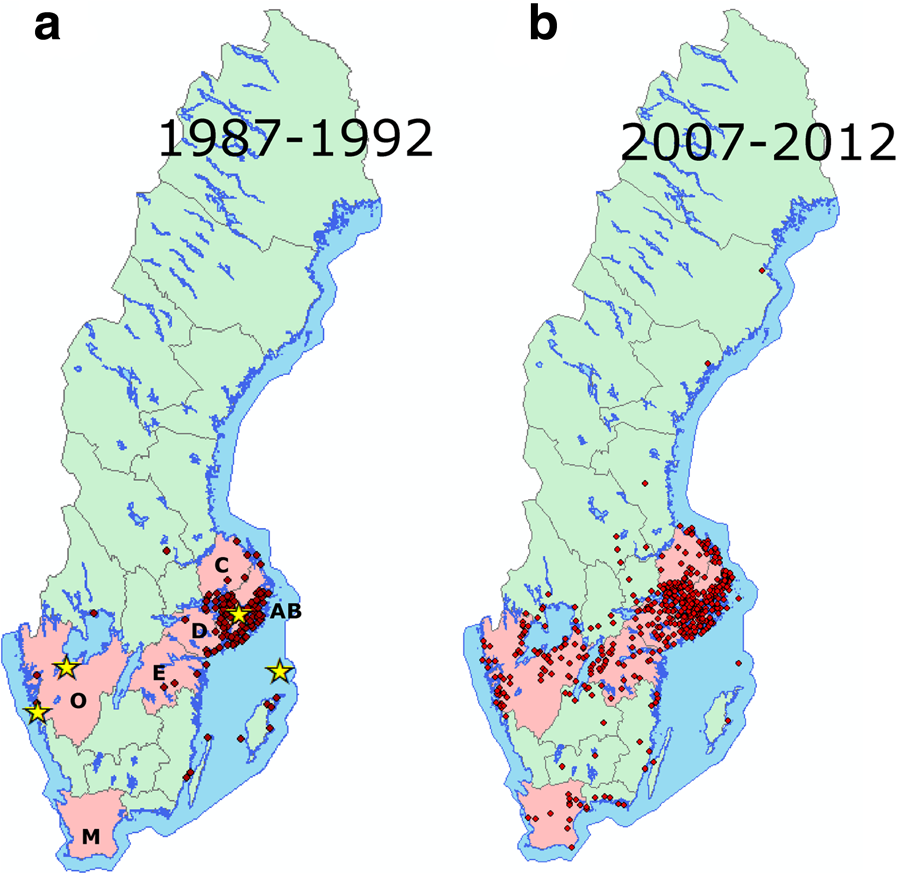
Each red dot on the above maps of Sweden represents a locality where one or more persons are presumed to have contracted TBE. The map to the left (**a**) shows the probable places of infection of all domestic human TBE cases with known place of infection (*n* = 308) recorded by the Swedish Institute for Communicable Disease Control (SMI) during the 6-year period 1987–1992. The map to the right (**b**) shows the corresponding data 20 years later, i.e. all domestically acquired human TBE cases with known place of infection (*n* = 1164) recorded during 2007–2012 (recorded by SMI and The Public Health Agency of Sweden). The light red areas on the maps represent the six counties that were analysed regarding wildlife abundance and TBE incidence. TBE data and wildlife hunting data from six counties (län) were analysed: Stockholms län (AB); Uppsala län (C); Södermanlands län (D); Östergötlands län (E); Västra Götalands län (O); and Skåne län (M). Each star denotes the location of a meteorological station that recorded data used in our analyses

The annual numbers of human cases of neuroinvasive TBE per 100,000 inhabitants were used as the dependent variable. We used TBE incidence data from six counties for the period 1986–2012. These TBE data were reported by the physicians treating the TBE patients and/or by the laboratories performing the analyses and if necessary completed by staff at the affected county medical office or at SMI (the Swedish Institute for Communicable Disease Control, which in January 2014 became the Public Health Agency of Sweden). The six counties (Fig. 3) encompass the four main TBE endemic counties in Sweden: Stockholms län (AB); Uppsala län (C); Södermanlands län (D); and Östergötlands län (E). AB, C, D and E are the letters used by each county government as a designation of its county. The fifth and sixth counties for which we used TBE data were the southernmost Swedish county, Skåne län (M) and Västra Götalands län (O). Several new TBE foci have appeared in these two counties during the last two decades.

### Data on the abundance of tick maintenance hosts and their predators

We used annual statistics on the numbers of seven different wildlife species, i.e. roe deer, Eurasian elk, red deer, fallow deer, wild boar, mountain hare (*Lepus timidus*), European hare (*L. europaeus*), harvested by hunters as proxies for the true densities of the wildlife species considered to be the most important maintenance hosts for *I. ricinus* in Sweden. We also included abundance data on their most important predators, the red fox and the lynx. The annual hunting statistics for the period 1960–2012 in the six regions were compiled by the Wildlife Monitoring Unit of the Swedish Association for Hunting and Wildlife Management. We consider these data to be the most useful and reliable data that exist concerning the relative densities of the roe deer, Eurasian elk, fallow deer, red deer, wild boar, mountain hare, European hare, red fox and lynx.

We assume that the annual numbers of mammals recorded as shot are directly proportional to their true densities. Support for this assumption comes from many studies [114] including a Swedish investigation by Andrén & Liberg [115]. They showed that hunting data on annual numbers of roe deer killed correlated with estimates of annual roe deer densities (*r*^2^ = 0.73, *n* = 44, *P* < 0.001). The latter variable had been estimated by pellet count surveys that should be directly related to the true density of roe deer [115]. Further support for the usefulness of hunting time series as a reliable proxy reflecting the annual variations in true density of certain game species is given by Cattadori et al. [114] and Jore et al. [61, 116]. Certainly, the temporal variability of game shot is influenced by many factors [117] such as hunting activity, which may differ among seasons and years. Thus, the number of active hunters, which may vary from one year to another, can influence the numbers of game animals shot. In order to correct for such a possible bias we obtained data available on annual numbers of hunting licenses issued in each Swedish county from the Swedish Environmental Protection Agency. Data were only available for eight years (2005/2006–2012/2013) and no information was available regarding in which county and for which game species a particular licence was used. The analyses revealed no significant correlation (Pearson’s correlations) for most of the pair-wise correlations, i.e., number of hunting licences issued and numbers of animals shot (European hare, fallow deer, lynx, Eurasian elk, red deer, red fox, roe deer) except for a negative correlation for wild boar (*r* = -0.337, *P* = 0.041) and a positive correlation for mountain hare (*r* = 0.569, *P* = 0.002). However, the numbers of killed animals of each species, except lynx and red fox, increased during the 8-year period. The numbers of hunting licences issued decreased during this period (*r* = -0.470, *P* = 0.0033). This suggests that these hunting licence data are of little or no value as a proxy for ‘hunting activity’ for our analysis on the potential relationship (s) between the abundance of large mammals and human TBE incidence.

### Temperature data

Temperature data were recorded by the Swedish Meteorological and Hydrological Institute (SMHI). Temperature readings were taken every third hour from 1st January 1961 to 31st July 2012 at four different localities (Fig. 3: Göteborg, Såtenäs, Stockholm and Gotska Sandön) in southern Sweden. The Stockholm station is located in the area of south-eastern Sweden where TBE cases have been continuously recorded since the disease was first discovered in Sweden in the 1950s. A few cases have been reported from the isolated Baltic island Gotska Sandön. The other two stations, Göteborg and Såtenäs, are located in south-western Sweden where TBE cases were only very rarely recorded before the end of the 1990s but where the incidence has since then increased. We consider the data recorded at these four stations to be acceptably representative for the study areas.

Change in mean temperatures was analysed using TableCurve software [118]. TableCurve contains more than 3600 built-in equations and automatically sort and plot the fitted equations according to chosen criteria. In this case we used transition functions, which define the total duration of a transition from one state or level to another.

The vegetation period is defined here as beginning with the first period of the year when the diel (24-hour) mean temperature is ≥ 5.0 °C, if this period is not followed by an equally long or longer continuous period with a diel mean temperature < 5.0 °C. The end of the vegetation period is calculated in a corresponding manner, i.e. the last day of the vegetation period is the day before the first 24-hour period with a mean temperature < 5.0 °C. Thus, the duration of the vegetation period is, in general, the number of days from the first day until the last day when the diel mean temperature is ≥ 5.0 °C.

Computation of the annual temperature sums were based on days with mean daily temperatures above 9 °C. All temperature data were rounded to the nearest degree, so that for example, values ​​in the range 9.5–10.4 °C became 10 °C. Subsequently, a temperature surplus was calculated, which is the difference between the current diel mean temperature and 9 °C. For example, if the rounded temperature is 9 °C or colder the temperature surplus is 0 (zero) °C and if the recorded temperature is 15 °C the temperature surplus is 6 °C. All such excess temperatures for one month were added and were treated as that month’s temperature sum. This sum was divided by the number of days in that month to give a monthly index. All monthly indices for that year were then added to create an annual temperature sum.

We also analysed, by calculation of the Pearson’s partial correlation coefficient, possible effects on human TBE incidence (number of human cases of neuroinvasive TBE per 100,000 inhabitants) by two other temperature-related parameters: (i) the annual mean temperature recorded in Stockholm (1969–2013); and (ii) the annual (1969–2013) mean temperature deviation from the ‘normal mean temperature’, which was calculated from the mean temperature for 1961–1990. These data were used for estimating an overall temperature change, but for the other analyses temperature data corresponding to the same period as for the TBE incidences and wildlife data were used. We tested the potential effect of no lag phase as well as a lag phase of 1, 2 or 3 years. The change in temperature sum was also tested with a transition function in order to reveal when the largest increase in temperature sum took place. Note that this was done on temperature sum as described above, not on annual mean temperatures.

### Statistical analyses

All statistical analyses were computed in SAS® statistical software version 9.3 TS Level 1MO. In order to achieve normal distributions the following variables were log-transformed prior to analyses: numbers of TBE cases related to human population size (100,000 inhabitants), numbers of animals shot, and duration (days) of the vegetation period. Likewise, the temperature sums were square-root-transformed.

Data for TBE incidences and number of killed animals were first standardised by region (county) in order to permit comparisons between regions by setting the mean for each region and variable to zero (but keeping the original variance). Thereafter the residuals were calculated, year being the independent variable. The residual data were analysed using PROC STATESPACE. The state space model represents a multivariate time series through auxiliary variables (state vectors), some of which may not be directly observable. The state vector summarises all the information from the present and past values of the time series. The observed time series are expressed as linear combinations of the state variables [119]. In the analyses the data for each game animal was compared to the incidences of human TBE and we specifically analysed (i) time series without any lag between the variables, and (ii) time series with lag = 1, i.e. covariance between values for number of killed animals at time t and incidence of human cases of TBE at time t+1 (one-year lag). Longer lags were also explored, but revealed no significant results. Finally, the t-values from the STATE SPACE analyses were summarised using meta-analysis. We used the software MetaWin [120] for that purpose. The same procedure was carried out to assess the potential impact of temperature sum and length of vegetation period on TBE incidence.

## Results

### Is the increase in TBE incidence due to wildlife abundance?

**Fig. 4:**
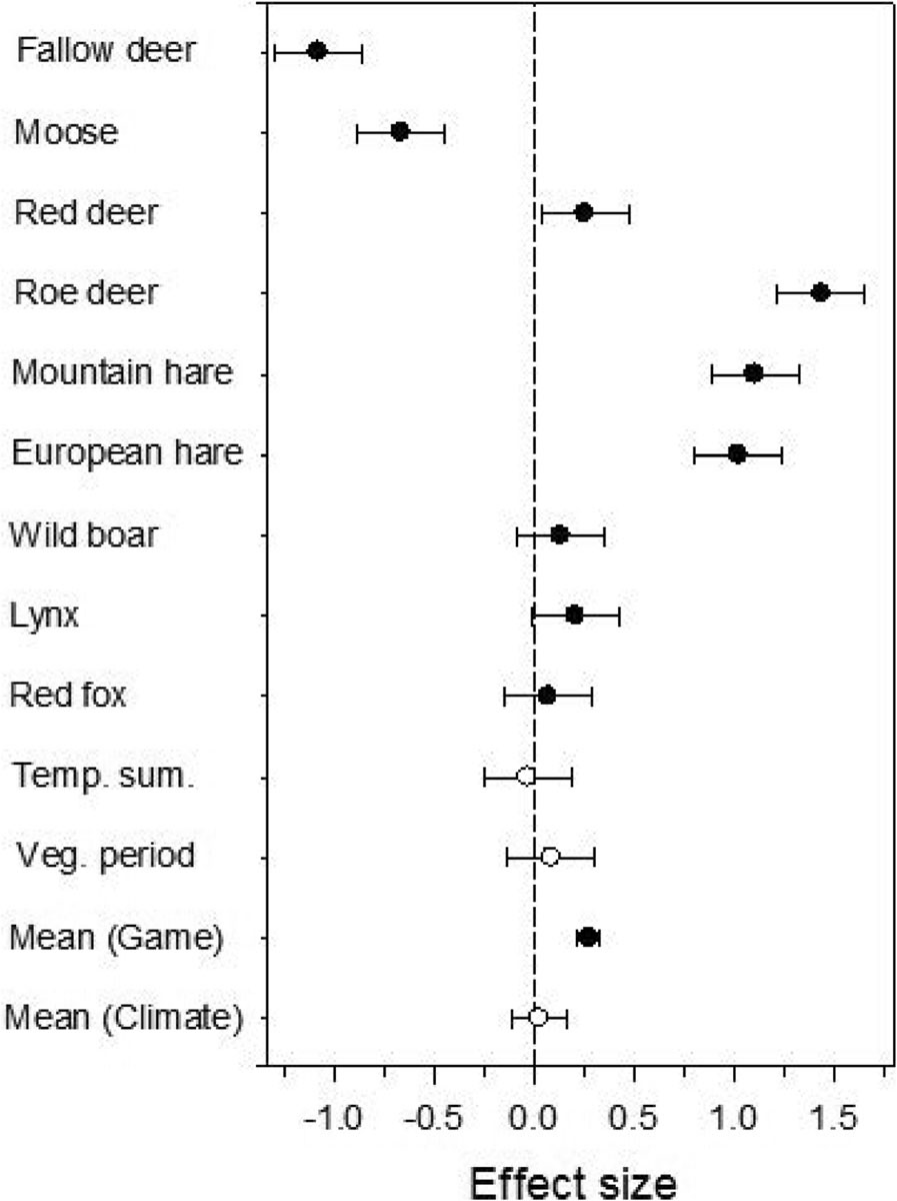
Meta-analysis of the effect of number of killed game animals (filled symbols) and climatic variables (open symbols) on incidences of TBE. For each entry (except the mean values) the values for six counties in Sweden were included. A positive value means that a high number of killed animals or high values of the environmental variables in one year results in increased numbers of cases of human TBE recorded the following year (i.e. a lag period of one year). The dots show the mean effect sizes, the bars the 95% confidence intervals. If the bars reach or cross the zero line it means that there was no significant effect of killed animals or environmental variables on TBE incidences. Fail-safe result: 20.4 (Orwin’s method [159]), meaning that 20.4 additional studies (in this case regions) are needed to reduce the observed mean effect size to a desired “minimal” effect size [159]. In this case the minimum effect size was set to 0.2 [160]

**Fig. 5:**
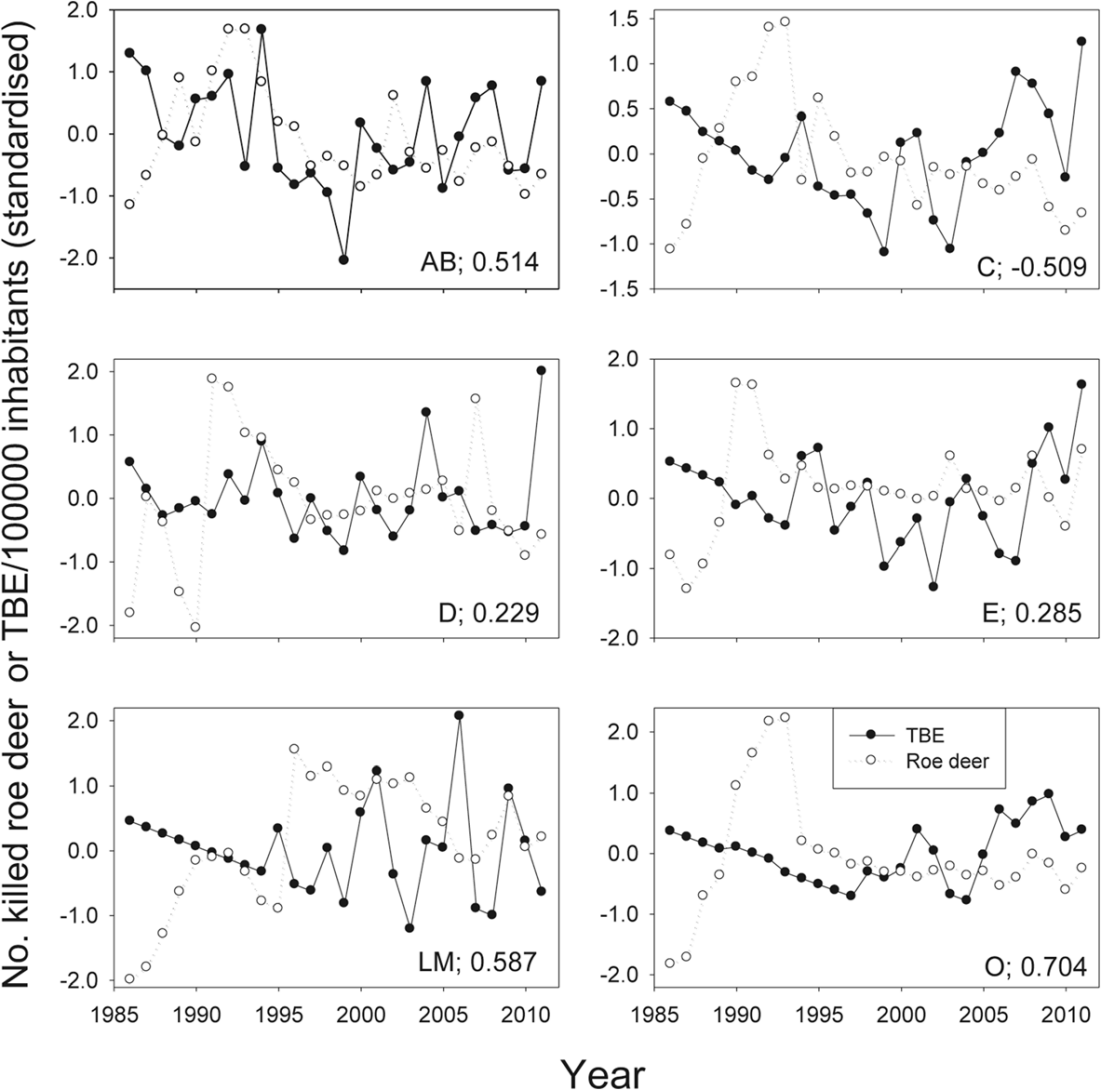
Standardised residuals for TBE incidences (number of TBE cases/100,000 inhabitants; filled symbols) and number of killed roe deer (open symbols). The letters in the bottom right of each figure indicate the county and the coefficient for the correlation between number of roe deer at time t and the TBE incidence at time t+1 year

When a time lag of one year was applied, the results showed that, on average, the red deer, roe deer, mountain hare and European hare exhibited positive, significant covariance; the Eurasian elk and fallow deer negative, significant covariance, whereas the wild boar, lynx and red fox showed no covariance with the incidence of human TBE (Fig. 4). When the analyses for all game animal species were combined, there was a significant correlation between the numbers of killed animals and the incidence of human TBE. Roe deer exhibited the highest significant association, although not significantly different from those of the mountain hare and European hare. The de-trended standardised values for roe deer abundance *versus* incidence of TBE are shown in Fig. 5 for each of the six regions included in this study. The covariance was positive in five of the regions and clearly significant in three (Fig. 5). The analyses for time series without a time lag of one year did not return any significant results. These results suggest that one or more factors, related to the abundance of roe deer, red deer, mountain hare and European hare explain the changes of the TBE incidence one year later. One or more factors related to roe deer abundance have the strongest impact on the variation in human TBE incidence.

### Is the increase in TBE incidence due to an increase in temperature?

**Fig. 6:**
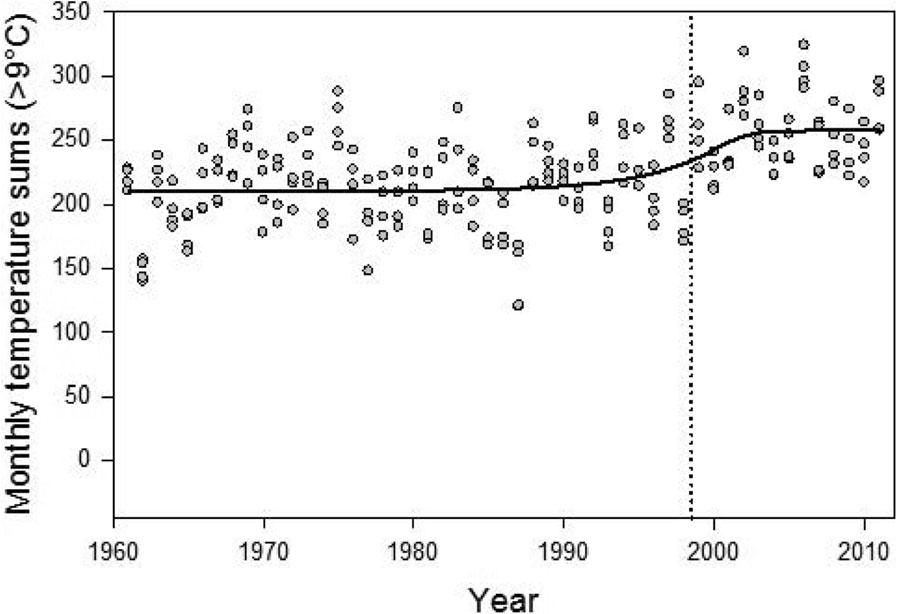
Transition function (an asymmetric sigmoid function) was applied to the temperature data (*r*^2^ = 0.266, *F*_(4, 199)_ = 18.01, *P* < 0.001). The transition centre, denoted by the dotted vertical line in the figure = 1998.5 and transition width = 5.3 years. This means that most of the increase in temperature sum took place between 1996 and 2001

The two climatic variables, annual vegetation period and annual temperature sum, did not exhibit any significant covariance with incidence of TBE, either for lag = 0 year or for lag = 1 year (Fig. 4). Based on the temperature measurements from four meteorological stations (Göteborg, Såtenäs, Stockholm and Gotska Sandön) in southern Sweden a transition function (Fig. 6) showed that the monthly sums of temperatures > 9 °C gradually increased from the mid-1990s until about 2002 when a new higher level was reached. Using temperature sum data only from the transition period (1995–2003) revealed no difference between sites over the years (*F*_(3, 20)_ = 1.86, *P* = 0.169) and no significant interaction between year and site (*F*_(3, 20)_ = 0.03, *P* = 0.991) with the years being a covariate in an ANCOVA. Thus, the increase in temperature did not differ between the sites between 1995 and 2003. There was, however, a time effect, i.e. the temperature increased over the same period (*F*_(1, 20)_ = 7.11, *P* = 0.013), as was already predicted from the transition function (Fig. 6).

Two time periods (1983–1994 and 2000–2012) were selected to represent the time before and after 1997, when the first TBE case was found to have been infected on the Swedish South-West Coast. Table 1 shows that the mean annual temperature sums differed among the meteorological stations (*F*_(3, 95)_ = 10.0, *P* < 0.001) and among the periods (*F*_(1, 95)_ = 56.31, *P* < 0.001) but there was no significant interaction among stations and periods (*F*_(3, 95)_ = 0.16, *P* = 0.922). The mean annual temperature sums were significantly higher at all four locations after 1999 than before 1995 (Table 1). Moreover, during the period 1983–1994 the mean annual temperature sums were similar at all four stations, i.e. the Stockholm and Gotska Sandön stations (representing the eastern, “old TBEV-endemic” region) and the Såtenäs and Göteborg stations. The latter stations represent the “new TBEV-endemic” region of south-western Sweden where human cases of TBE were almost never recorded before 1997.

**Table 1:** Analysis of temperature sums recorded at four meteorological stations from 1983–1994 and 2000–2012

Station	1983–1994	2000–2012	*P*-value*
Stockholm	228.8 ± 32.01^a^	273.9 ± 27.03^b^	0.0017
Gotska Sandön	195.3 ± 30.21^a^	241.3 ± 25.26^a^	0.0071
Såtenäs	193.2 ± 33.60^a^	240.3 ± 22.90^a^	0.0070
Göteborg	227.8 ± 29.07^a^	240.3 ± 22.90^a^	0.0300

^*^Level of significance between periods within stations

The column to the right shows the level of significance between periods within stations. Means within columns denoted with the same superscript letter (either ^a^ or ^b^) are not significantly different at *P* = 0.05

Hypothetically, an increase in the environmental temperature might increase the questing and blood-feeding activity of ticks, the rate of multiplication of TBEV in infected ticks and the rate of transmission from infective ticks to susceptible humans. A higher temperature might also affect the transmission rate by increasing the contact rate between ticks and susceptible people (including persons without adequate levels of anti-TBEV antibodies due to inadequate vaccination) who then spend more time outdoors in “TBE risk areas”. *Ixodes ricinus* ticks usually become infected with TBEV in the larval stage and may, after one or more years as infective nymphs transmit the virus to humans. Thus, it is plausible that the temperature in the tick’s environment may affect the human TBE incidence after such a lag period of one or a few years. Therefore, in order to make the analysis all-inclusive we complemented the previous analyses by investigating (i) the deviation of the annual mean temperature from the annual mean temperature for the normal period 1961–1990; and/or (ii) if the annual mean temperature may have affected the annual incidence of human, neuroinvasive TBE for 1969–2013. Pearson’s correlation coefficient was computed for (i) the deviation of the annual mean temperature and the annual incidence of human TBE, and (ii) the annual mean temperature and the annual incidence of human TBE. Both were computed for each weather station. None of the eight pair-wise correlations was statistically significant. This suggests that one or more factors, not directly related to temperature, better explain the changes of the TBE incidence.

## Discussion

Our results show that abundances of roe deer, red deer, mountain hare and European hare correlate positively with the incidence of human TBE. One year after increased density of any one of these animal species there is, *in general*, a significant increase of the TBE incidence. Among these animal species just mentioned the number of roe deer shot has the strongest correlation with the TBE incidence one year later.

The mean duration of development from egg to nymph and from egg to adult of *I. ricinus* in southern Sweden is generally considered to be about two years and about three years, respectively [22, 40]. If correct, and since it is usually the nymphs, which transmit the TBEV to humans one would expect the lag period between peak roe deer density and peak TBE incidence to be about two years rather than one year. There are two plausible explanations for this seemingly paradoxical result.

First, the majority of roe deer shot are bucks older than one year [121]. They therefore represent a segment of the roe deer population, which potentially has provided blood to the tick population for more than one year. This suggests that the real lag period is longer than one year, which would agree with a three-year life-cycle of *I. ricinus* in southern Sweden.

Secondly, in southern Sweden an unknown proportion of the nymphs may possibly be younger than two years. We are not aware of any field experiment performed in southern Sweden that provides evidence that the mean duration of development from egg to adult of *I. ricinus* is about three years. A reason for a life-cycle shorter than three years could be that the microclimate in the densely tick-infested biotopes and in particular in the TBEV foci of southern Sweden, are in general warmer than the surrounding environment [22, 122]. Thus, a generally higher temperature in the densely tick-infested woodland and surrounding ecotones, especially the TBEV foci would promote co-feeding transmission of the virus and speed up the development of both virus and its tick host. Moreover, in the warmer, more favourable locality the host density may be higher than in the surroundings. High availability of different hosts for the ticks would also increase tick development and tick survival.

A main reason for the increased density of ticks and consequently the increased incidence of human cases of TBE during the last two decades is most likely that the number of tick maintenance hosts, i.e. the biomass of suitable tick hosts, especially roe deer [59], has been at a considerably higher level since the 1980s than it was during and before the mid-1900s [59] (Fig. 2). Even though the abundance of roe deer has declined since the mid-1990s it is still much higher than before the 1980s [59] (Fig. 2). In many biotopes suitable for *I. ricinus,* the roe deer is still the most important tick maintenance host. But in many parts of southern and central Sweden the red deer and fallow deer are becoming increasingly important as tick maintenance hosts [58]. Another factor, which most likely has had a great impact on the TBE incidence, is that deer are now abundant in potential tick habitats where previously tick maintenance hosts were absent or rare. The role of deer in these areas has therefore changed from being a limiting factor for the tick population to permitting an almost unlimited rate of increase of the tick population. In these areas it is presumably the availability of small mammals, i.e. the main hosts of the tick larvae, which are now limiting the increase of the tick population [38].

The results from Uppsala County did not conform to those of the other counties. In some areas of Uppsala County the predation pressure by lynx has been so strong during the last two decades that roe deer have disappeared [59]. This could be the explanation for the lack of correlation between roe deer abundance and TBE incidence in Uppsala County during recent years.

The data presented here is another example of the many factors, which may influence the incidence of human TBE. Several of these variables are interacting and will vary in strength with time and among different localities. The end result on the human TBE incidence can therefore be exceedingly difficult or impossible to predict. See Additional file 1 for other factors, which may affect the human TBE incidence.

It needs to be emphasised that there are obvious exceptions to the generalisation that “high tick abundance is due to high abundance or presence of roe deer”. For instance, in Norway, high tick abundance is correlated with high densities of red deer and farm animals, not to numbers of roe deer [116]; in Sweden on the Baltic island of Gotska Sandön the mountain hare is the only maintenance host for the abundant tick population there [123, 124]; and in South Wales, UK, high tick densities were related to grazing livestock on rough grassland where deer were absent [125]. These examples show that the function of roe deer as an important tick maintenance host can be replaced by other ungulates and even by hares.

Hofmeester et al. [34, 35] recently showed that in the Netherland the density of *I. ricinus* was significantly higher in 17 one-hectare study plots than in in 3 one-hectare plots without deer*.* They also showed that any further increase of host density above a relatively low threshold density did not increase tick density [34, 35].

Our study revealed a significant positive correlation between roe deer abundance and TBE incidence, which we regard as a proxy for tick density. Our results do not contradict those of Hofmeester et al. [34, 35]. The explanation is that our Swedish data refer to large regions (counties) of many km^2^ whereas the data collected in the Netherlands were from much smaller, one-hectare plots [78]. In Sweden, before the mid-1980s many biotopes potentially suitable for both deer and ticks were free of these organisms. With the expansion of the roe deer population during about 1985–1995 many young roe deer left their places of birth to search for new deer-free biotopes [126]. Most or all of these migrating young roe deer were presumably, during most of the year, tick-infested. Thereby even the ticks became distributed to new localities. As a consequence most or all woodland biotopes in southern Sweden became inhabited by deer [59] and ticks [38]. This increasingly larger tick-inhabited area resulted in a successively greater number of potential TBEV foci. This explains the positive correlation between roe deer abundance and TBE incidence until about 1995.

### How are TBEV foci established?

The increased density of potential vectors is likely to have promoted and still favours transmission of the TBE virus in such recently created TBEV foci. Thus, more roe deer in south-western and southern Sweden increased the number of TBEV foci, which increased the risk for people in these regions to become infected with TBEV. This also explains why human TBE cases began to appear in western and southern Sweden in the late 1990s to early 2000s. When the density of potential vectors increased so did the potential of virus transmission and the likelihood that the virus became more prevalent in the tick population. This is likely to have favoured transmission of TBEV to humans. With an increasing number of human TBEV infections, a high proportion of which are without clinical symptoms, the number of clinically apparent TBE cases increased (Fig. 1).

The spread of TBEV and establishment of new TBEV foci may take place by TBEV-infected viraemic rodents, hares and insectivores, and by several species of virus-infected ground-frequenting birds. The virus may also spread to new areas by birds infested with TBEV-infected ticks as well as by small and larger mammals infested with such ticks. For instance, if a massively tick-infested, large mammal migrates away from its place of birth - hypothetically a TBEV-enzootic area - to another locality where the tick-infested mammal or bird stays or dies, a new TBEV-focus may appear. This is because some of the ticks brought to the new locality might have been infected with TBEV at the original TBEV focus. Other, non-infected ticks may then become infected either viraemically from the infected host or non-viraemically from infected ticks co-feeding via the host, or by both means. After death of the non-immune host numerous virus-infected ticks may have metamorphosed to host-seeking virus-infected nymphs or adult ticks.

Among larger mammals we consider the roe deer to be a good candidate for the long-distance spread of TBEV-infected *I. ricinus*, which may become the founders of new permanent TBEV foci. This is because roe deer are commonly infested by all active stages of this tick species [25] and because the young roe deer bucks often undertake extensive migrations [126].

It could be that occasionally small TBEV foci are formed in a manner similar to that by which some foci of another *Flavivirus*, the Kyasanur Forest disease virus (KFDV) foci are thought to arise [127, 128]: larvae and nymphs of the main KFDV vector *Haemaphysalis spinigera* happen to infest a viraemic, dying monkey. Later, after death of the monkey, when the tick larvae and nymphs have metamorphosed into nymphs and adults, respectively, the spot where the monkey died may harbour numerous questing, virus-infected ticks. They will attempt to attach to any potential vertebrate host passing through this incipient KFDV focus, which was originally “established” by the death of a tick-infested monkey. A similar way of establishment of KFDV foci may involve not only monkeys but any other KFDV-viraemic mammal such as a deer species. Young roe deer fawns, without any effective protective immunity to pathogens such as the TBEV, can be very heavily infested with all active stages of *I. ricinus.* Therefore, we consider such animals to be “almost optimal candidates” for the effective formation of TBEV foci in TBEV-enzootic regions. The fact that transovarial transmission occurs to some degree with TBEV in *Ixodes* ticks might render this virus very likely to become established and to occur in geographically small virus foci in nature.

### How can we explain the increasing TBE incidence simultaneously with the declining roe deer population after 1995?

The Swedish roe deer population rose to a marked peak in the late 1980s and early 1990s, where after the level gradually decreased. Yet, it has remained much higher than it was before the 1980s (Fig. 2). Even one or a few deer can sustain a relatively dense tick population provided the habitat is not too large [35]. Another factor to realise is that if the density of deer decreases the availability of potential hosts to host-seeking ticks is consequently reduced. They will therefore be diverted to feed on other hosts, many of which may be competent TBEV-transmission hosts, and on humans [72, 129]. The diversion of a greater segment of the immature tick population to feed on transmission-competent hosts will likely increase the density of TBEV-infective ticks. Thus, when deer abundance declines from a high level the result may be an increased incidence of human TBE [40, 130]. The TBE risk becomes even stronger when particular weather conditions increase the number of people and the time they spend picking berries or mushrooms in woodland habitats potentially infested with TBEV-infected ticks [40, 130]. An additional explanation is that, during recent decades, other large ungulates such as red deer [58], fallow deer [131] and wild boar [132], of which at least the red deer is an efficient tick maintenance hosts, have increased their population sizes both in regions where roe deer numbers have decreased and in other areas. Thereby, the number of ticks has been maintained or even increased in southern Sweden.

The likelihood that ticks will mate and that tick females will become inseminated depends, indirectly, on host density [133]. Thus, a sparse population of *I. ricinus,* feeding and mating on a high-density host population, may not increase in numbers due to insufficient mating and insemination [133]. This important phenomenon shows that the deliberate reduction of the density of an important tick maintenance host may not always be desirable. Instead, reducing the density of such a tick host may increase the ticks’ mate-finding success and thus the fecundity of the tick population. This conforms to our findings; the incidence of TBE continued to increase up until 2013 (Fig. 1) despite the decline of the roe deer population after the peak in the early 1990s (Fig. 2) due mainly to increased predation by lynx, red fox and hunting. It also supports the notion that the densities of tick maintenance hosts need to be reduced to very low levels; otherwise the abundance of ticks will remain at a high level and transmission of TBDs will continue at unacceptable rates. The previous examples illustrate that the relationship between density of the tick’s maintenance hosts and tick density is not always linear [35, 72, 133].

Another factor which may have contributed to the increasing TBE incidence is climate change by which the increasing temperature in the ticks’ environment is likely to have increased the rate of development of both the virus reservoir, i.e. the tick, and the virus.

### Increased density of tick hosts may lead to increased tick densities and increased transmission of tick-borne pathogens

Increased abundance of potential vectors is often related to an increased risk of spread of arthropod-borne pathogens [134, 135]. Well-known examples of this epidemiological phenomenon are the high densities of the white-tailed deer (*Odocoileus virginianus*) in North America which have indirectly dramatically increased the densities of *I. scapularis* and *Amblyomma americanum.* The high abundances of these tick species have then increased the incidences and geographical distributions of *I. scapularis*- and *A. americanum-*associated zoonotic pathogens [136]. The main vectors of *B. burgdorferi* in North America are usually not found at high densities in the absence of deer [137, 138]. And there is strong evidence that the abundance of white-tailed deer determines the abundance of *I. scapularis* in north-eastern USA [137, 139–141].

The density of *I. ricinus* is dependent on climatic conditions and availability of vertebrate hosts [74]. Local densities of *I. ricinus* are often - but not always [35] - positively correlated with those of deer except at very low and very high deer densities [142, 143]. This is because deer are, in most North-European localities, keystone species of outstanding importance as maintenance hosts for the adult ticks of *I. ricinus* [25]. Deer are also very important hosts for the larvae and nymphs, although small mammals and birds are usually more important than deer as hosts for the tick larvae [25, 74]. Therefore, these smaller vertebrates may, in comparison to deer and other larger tick hosts, be more important in limiting tick density. However, in ecosystems with a less diverse fauna of vertebrates, such as in Scotland, a greater proportion of the immature segment of the tick population may feed on larger vertebrates, e.g. deer. This implies that in the latter situation, i.e. in ecosystems where most tick larvae will feed on deer, there will be a stronger correlation between deer density and tick density.

Important investigations on the relationships between deer abundance and climatic factors as determinants of *I. ricinus* density and *B. burgdorferi* (*s.l.*) prevalence have been carried out in Scotland [60, 74, 144]. Some of the results are based on studies of deer exclosures on moorland or in forest, at a range of different deer densities and provide powerful evidence that areas with fewer deer have fewer ticks, and that both large and even very small fenced exclosures have significantly lower numbers of nymphs: fencing or reducing deer density by culling will both dramatically reduce tick abundance, equivalent to 86–96% control of questing nymphs [60]. On the island of Funen, Denmark, a major epizootic among roe deer seems to have drastically reduced tick abundance with the effect that the incidence of human neuroborreliosis declined significantly [21].

In agreement with the Swedish studies [145] James and colleagues also found that in Scotland nymphs are more likely to be infected with *B. burgdorferi* (*s.l.*) where red deer densities are higher [74] despite the fact that deer is an almost incompetent reservoir for *B. burgdorferi* (*s.l.*) [33]. The explanation is similar to that for the Swedish results: red deer also feed large numbers of adult tick females, which produce vast numbers of offspring. Most of the resultant tick larvae will feed on small ground-frequenting vertebrates (shrews, rodents, hares, birds), which in general are competent transmission hosts for one or more of the *B. burgdorferi* (*s.l.*) genospecies. This will increase the probability of spirochaetal transmission from such vertebrates to immature ticks and from co-feeding *Borrelia-*infected to infectible immature ticks [74]. Only at a very high availability of red deer in relation to that of small mammalian transmission hosts will the so-called dilution effect occur.

In agreement with the situation just described about a relationship between deer abundance and density of *B. burgdorferi* (*s.l.*)-infected ticks, similar models of transmission of Louping ill virus in Scotland [144] and of TBEV in Italy [71] have been proposed: the prevalence of these viruses in the tick populations are expected to increase with increasing deer densities. Only at very high deer densities will the dilution effect become obvious [71, 73, 74, 144].

Several other investigations also support the notion that the incidence of human TBE is related to the density of deer [68]. Thus, in the Czech Republic, Zeman & Januska [82] showed that the risk that people will contract a TBEV infection is associated with the abundances of roe deer and mice (*Apodemus* spp.). In Italy, Hudson et al. [77] provided further evidence that roe deer are important for the maintenance of a high tick density and therefore, indirectly, for transmission of the TBE virus. More recently, Knap & Avšič-Županc [75], working in Slovenia, showed that the TBE incidence correlates geographically with red deer abundance. Another important recent discovery was done by Jemersic et al. [146] who demonstrated the presence of TBEV in the blood of red deer. Thus, in TBEV-endemic areas roe deer and red deer abundances may be useful indicators of the risk that people may contract a TBEV infection.

In a recent study Kriz et al. [147] examined the potential contribution of the two most widespread game species, roe deer and wild boar, to the high incidence of TBE in the Czech Republic. They used the annual numbers of game shot as a proxy for the densities of different wildlife species. Statistical analyses suggest that the size of the wild boar population contributed to the presently high and increasing incidence of TBE. In contrast, the regulated population of roe deer appeared not to have significantly contributed to the recent geographical and temporal increase of human TBE cases in the Czech Republic [147]. Thus, in these respects the situation in the Czech Republic differs from that in the six Swedish counties analysed in the present paper. Also in Finland the incidence and geographical distribution of human TBE cases and the appearance of new TBE foci appear to be related to the increased ranges of roe deer and white-tailed deer populations [148].

Numerous studies have provided strong evidence for the important role that certain ungulates, particularly some species of wild cervids play as tick maintenance hosts, since they are preferred hosts for the adult ticks. Reducing the availability of such vertebrates to ticks can have profound, negative impact on tick populations and on reducing the incidence of TBDs [8, 11, 60, 63, 64, 129, 137, 139, 141, 149].

### Does the warmer climate affect TBE incidence?

The mean annual temperature recorded at most European meteorological stations, increased suddenly at the end of the 1980s [38, 72, 150]. The mean annual temperature has then been followed by consistently warm conditions [38, 72, 150]. Such an increased warming, most of which occurred from January to early August [72] should both directly and indirectly have favoured survival and reproduction of both roe deer and ticks [38]. In Sweden the higher annual mean temperatures since 1987–1988 [150] are reflected in our data by a longer vegetation period and an increasing temperature sum. Even if these parameters, in our analyses, were not significantly correlated with the incidence of human TBE, they are still considered to be favourable for the tick population in many regions of Sweden [38, 105]. Provided that the precipitation is adequate for the ticks’ survival, an extended vegetation period, with earlier springs and later autumns, will certainly increase the time available for the ticks’ host-seeking and other activities. This would increase the fecundity resulting in a more rapid growth of the tick population. A longer growing season is, in general, also favourable for the winter survival of the ticks’ primary maintenance host, the roe deer [126], which thereby would also favour the growth of the tick population indirectly [40, 105]. Based on a seven-year long field study in France, Paul et al. [151] concluded that despite complex intra-annual relationships between tick densities and temperature, there was no evidence for a climate-associated increase in risk of infection with any of several tick-borne pathogens. Rather, tick densities are mainly determined by the density variations of the vertebrate tick hosts. However, the authors emphasised that further research on the impact of climate change on tick population densities is needed [151].

The ongoing climate change is affecting the seasonal activity patterns of larvae and nymphs of *I. ricinus* so that in certain areas the seasonal activity patterns of these segments of the tick population will become more synchronous. In localities where susceptible tick larvae and infective nymphs often feed close together on the same host, co-feeding transmission of TBEV is facilitated and will potentially increase the density of TBEV-infective ticks [152]. This phenomenon may be an additional factor, together with increased numbers of tick maintenance hosts, which may explain the appearance of new TBE foci in previously “non-endemic areas”. However, the distinct increase of the incidence of human TBE in south-western Sweden did not occur until the first decade of the 2000s (Fig. 3; [40, 153]). The mean temperature recorded by the four Swedish meteorological stations increased throughout the period from 1960 to 2012. In addition, the temperature increase, measured as number of days with a temperature > 9 °C, did not differ among the four areas (Fig. 6). These results indicate that the trend of an increasing temperature during the last five decades is not the main reason for the appearance of TBEV in south-western Sweden.

Even though temperature is one of several contributing factors in the epidemiology of TBE [16, 105, 154], this parameter is presumably not the main factor explaining why new TBE foci have appeared mainly in south-western Sweden during the last two decades. The increase in human TBE incidence in this region presumably reflects a significant increase in density and distribution of TBEV-infected ticks, which resulted in increased virus transmission to humans. This was presumably mainly an indirect consequence of the significant increase in the abundance of roe deer and red deer leading to a significantly increased tick distribution and density with presence of ticks in biotopes, which had previously been tick-free. The presence of TBEV-infected ticks became apparent only when human cases of TBE were recorded in areas where human TBE had never before been diagnosed.

### Several interacting factors affect the ecology and epidemiology of TBEV

The role of roe deer and red deer as important maintenance hosts for *I. ricinus*, markedly affecting the epidemiology and incidence of TBE and LB in Europe, resembles in many respects the impact of white-tailed deer on LB incidence in North America [11, 155]. The increase of TBE incidence in humans in southern Sweden is most likely, to a great extent a result of the high abundance of tick maintenance hosts, in particular roe deer. Apart from high roe deer abundance, even red deer [58] and hare abundances [156, 157] were shown to be associated with human TBE incidence. Fallow deer and cattle exhibit comparatively sedentary habits and a tendency to form dense herds [131] therebydestroying the ground vegetation. Therefore, fallow deer and cattle often seem to reduce tick abundance [33] and TBE incidence. The abundance of small mammals, especially *Apodemus* spp., which act as hosts for non-viraemic and short-term viraemic transmission of TBEV to immature *I. ricinus*, most likely partly determines the infection prevalence of the nymphs about 1–2 years after peak abundance of the small mammal population. The important tick maintenance hosts, particularly roe deer, red deer, mountain hare and European hare have the potential to increase the density of all active stages of *I. ricinus*. These medium or large mammals, together with the small mammals, the latter which serve both as TBEV transmission hosts and food for immature ticks, are the most likely factors, determining the density of TBEV-infected ticks. The recent appearance of TBEV in south-western and southern Sweden is presumably a consequence of the relatively high abundance of cervids [59], which has now prevailed there for more than two decades. The warmer climate is favourable in several ways to survival and reproduction of both small and large mammals. Thus, the warmer climate may have contributed indirectly and will - in areas where precipitation will not decrease drastically - continue to increase the abundance of ticks and therefore also to increase the density of TBEV-infected ticks. The warmer climate has prolonged the tick season and possibly the time that people spend outdoors in biotopes potentially harbouring TBEV-infected ticks. Statistics Sweden report that both men and women increased significantly their outdoor activity, particularly walks in the woods, during the 1990s [158]. This may have increased the risk for people being exposed to ticks and to become infected with TBEV. The epidemiology of multi-component, vector-borne zoonoses is complex. Regarding the TBE virus it involves several virus subtypes, some of which may occur in the same limited focus. The virus interacts with one or more tick species, which function as virus reservoirs and virus vectors. The virus and the ticks interact with one or several vertebrate species, which may play the role as virus reservoirs and transmission hosts, and as hosts for immature ticks, sometimes also as hosts for adult ticks. One or more of these vertebrate species may function as tick maintenance hosts, which feed the adult ticks, and as hosts for immature ticks, and possibly also as virus transmission hosts for co-feeding ticks. Other vertebrate species, which may also function as tick maintenance hosts may not be competent virus transmission hosts. Under exceptional conditions such tick hosts will “dilute” the infection. The epidemiological picture of such a complex ecological system is not static. It is emphasised that the picture given in this paper is based on data collected during a certain time period in certain regions in southern Sweden. The relative importance, i.e. the impact by different organisms on the TBEV transmission intensity will certainly change with time and will differ from one geographical location to another.

## Conclusions

The increased availability of deer to *I. ricinus* over large areas of potential tick habitats in southern Sweden during the 1980s and early 1990s increased the density and range of *I. ricinus* and created new TBEV foci, which at least partly may explain why the incidence of human TBE has increased in Sweden during the last two decades.

## Additional file

Additional file 1: **Table S1.** Several interacting factors can affect the TBE incidence. (DOCX 22 kb)

## Abbreviations

LB: Lyme borreliosis
SMHI: The Swedish Meteorological and Hydrological Institute
SMI: The Swedish Institute for Communicable Disease Control, which in January 2014 became the Public Health Agency of Sweden
TBE: Tick-borne encephalitis
TBEV: Tick-borne encephalitis virus
