# Supplementary material for: The importance of wildlife in the ecology and epidemiology of the TBE virus in Sweden: incidence of human TBE correlates with abundance of deer and hares
Source: Parasit Vectors. 2018 Aug 29;11:477. doi: 10.1186/s13071-018-3057-4 (PMC6114827; doi:10.1186/s13071-018-3057-4)
Supplement: Supplementary file 1 — Table S1. Several interacting factors can affect the TBE incidence. (DOCX 22 kb) [file 13071_2018_3057_MOESM1_ESM.docx]

**Additional file 1**

**Several interacting factors can affect the TBE incidence**

Despite a drastically increased vaccination rate against TBE in Sweden since the late 1980s, the incidence of TBE has increased significantly during the same period up until 2017 [1]. There are at least six plausible major reasons (listed in bold style below) for the increasing TBE incidence since the year 2000 in Sweden:

**(i) Vaccination.** Many people who lived in or visited risk areas were not ‒ or were inadequately ‒ vaccinated against TBE. One among several variables that could influence the analyses is the vaccination frequency. Despite a drastically increased vaccination rate against TBE in Sweden since the late 1980s, the incidence of TBE has increased significantly during the same period up until 2017. The first TBE vaccine was approved for general use in Sweden in 1988. In the early 1990s some 10,000 doses were sold each year to Swedish customers. In the mid-1990s vaccination against TBE increased, and reached about 200,000 doses annually by the early 2000s. In 2013, 550,000 doses were sold in Sweden. Consequently, since 1990 there has been a >10–15-fold increase in the sale of TBE vaccine to Swedish customers. During the last 10 years the sale of the vaccine more than doubled (Dr Rolf Gustafson, M.D., Medical Director, Baxter Medical AB, Stockholm, Sweden, personal communication). The amount of vaccine sold is presumably proportional to the amount of vaccine used. There are no national statistics on how many persons are vaccinated against TBE annually. However, a study in the Stockholm area 2015 showed that more than 50 percent of the participants were vaccinated. Despite a relatively high vaccination rate, it is still insufficient; yet, 80-100 people are still reported to have been infected in the Stockholm region each year [1]. It is possible that to a certain extent the "wrong" segment of the population had been vaccinated. If people who rarely or never visited TBEV-enzootic areas became vaccinated while many people living in TBE risk areas remained without adequate vaccination such a phenomenon could partly explain the increased TBE incidence. However, we consider it likely that the increasingly more effective information in Sweden about ticks, TBDs and TBE risk areas, and the increasing TBE vaccination rate of Swedish people since the late 1980s has had as its main effect to prevent many vaccinated people to become TBEV-infected from ticks. Thus, without this high vaccination rate the TBE incidence would, most likely, have been much higher.

**(ii) The contact rate between susceptible humans and infective ticks.** The contact rate between infective ticks and susceptible people, i.e. persons not adequately vaccinated against TBEV infection, is an important factor that will influence the incidence of human TBE in time and space [2]. The general opinion is that people in Sweden spend more time in nature nowadays compared to some decades ago. One investigation performed by Statistics Sweden support this notion: adult women and men in Sweden used significantly more of their leisure time walking in the forests in 2002 than in 1976 [3]

**(iii) Number of potential vectors.** The density of infected ticks, particularly infected nymphs, is an important index of risk of transmission of TBEV to humans. Data suggest that the number of ticks as well as their geographical distribution in Sweden increased substantially during the last two to three decades [4]. This may explain much of the increased TBE incidence in Sweden up until 2013. The present study suggests that increasing numbers of tick maintenance hosts leads to increased tick abundance and increased density of virus-infected potential vectors, which facilitates increased transmission of the TBE virus.

**(iv) Prevalence of TBEV infection in the tick population.** Information available [5] is not sufficient to enable us to detect if the TBEV infection prevalence in the tick population changed significantly during the last decades.

**(v)** **Public knowledge and awareness about ticks and tick-borne diseases.** General practitioners as well as the general public are now, in general, more well-informed about tick biology as well as epidemiology and symptomatology of TBDs than 2-3 decades ago.

**(vi) Diagnostic methods and capacity for correct diagnosis of the TBEV infection**s. The methods and equipment available for correct diagnosis of TBE, Lyme borrelioses and a few other TBDs have become more reliable and more widely used.

In summary, there is presumably a combination of several of the listed factors, which may have affected the recorded and the true incidences of TBE and other tick-transmitted diseases of humans in Sweden during the last two to three decades.

**References**

1.        Folkhälsomyndigheten. Tick Borne Encephalitis (TBE). <https://www.folkhalsomyndigheten.se/folkhalsorapportering-statistik/statistikdatabaser-och-visualisering/sjukdomsstatistik/tick-borne-encephalitis-tbe/>. Accessed 20 Feb 2018.

2.         Randolph S. Predicting the risk of tick-borne diseases. Int J Med Microbiol. 2002;291 Suppl 33:6-10.

3.         Statistiska centralbyrån (SCB). Statistics Sweden: Fritid 1976-2002. Leisure activities 1976-2002. Stockholm 2004. https://www.scb.se/statistik/LE/LE0101/1976I02/LE0101_1976I02_BR_LE103SA0401.pdf. Accessed 15 Mar 2018.

4.         Jaenson TG, Jaenson DG, Eisen L, Petersson E, Lindgren E. Changes in the geographical distribution and abundance of the tick *Ixodes ricinus* during the past 30 years in Sweden. Parasit Vectors 2012;5:8.

5.         Pettersson JH, Golovljova I, Vene S, Jaenson TG. Prevalence of tick-borne encephalitis virus in *Ixodes ricinus* ticks in northern Europe with particular reference to Southern Sweden. Parasit Vectors 2014;7:102.
